# Analytical and experimental analysis of concrete temperature and energy considering open-air environmental variations

**DOI:** 10.1038/s41598-024-64568-6

**Published:** 2024-06-12

**Authors:** Wen-Jian Yang, Peng Li, Li Zhuo, Ming-Liang Pang, Hong-Qiang Xie, Ming-Li Xiao

**Affiliations:** 1 PowerChina Chengdu Engineering Corporation Limited, Chengdu, 610072 Sichuan China; 2 State Key Laboratory of Hydraulics and Mountain River Engineering, College of Water Resource and Hydropower, Sichuan University, Chengdu, 610065 Sichuan China; 3 Technological Innovation Center of Hydropower, Wind, Solar and Energy Storage of Tibet Autonomous Region, Chengdu, 611130 Sichuan China

**Keywords:** Concrete temperature, Analytical model, Environmental factors, Solar radiation, Longwave radiation, Engineering, Physics

## Abstract

Longwave radiation is an important open-air environmental factor that can significantly affect the temperature of concrete, but it has often been ignored in the temperature analysis of open-air concrete structures. In this article, an improved analytical model of concrete temperature was proposed by considering solar radiation, thermal convection, thermal conduction and especially longwave radiation. Temperature monitoring of an open-air concrete block was carried out to verify the proposed model and analyze the heat energy characteristics of open-air concrete. As demonstrated by the open-air experiment, under the influence of longwave radiation, the temperature at the top of the concrete block could decrease rapidly at night and even become lower than the minimum temperature at its bottom. Compared with the analytical model that ignores longwave radiation, the improved model that includes it better matches the measured temperature. According to the energy analysis, although solar radiation controls the transient variation in heat energy, the heat exchange caused by longwave radiation were more than that caused by convection on sunlit surfaces, which indicates the importance of considering longwave radiation.

## Introduction

Concrete is widely used in various engineering projects, and the temperature field can essentially affect the safe operation of concrete structures. Underestimating temperature loads during the design phase may lead to serious accidents, such as the buckling accident of the Danube 4th Bridge in Vienna due to the neglect of temperature load^1^ and the deck overturning accident of the Huaqiang North Interchange in Shenzhen due to the displacement accumulation caused by temperature changes^2^. Especially for thin-walled concrete structures, temperature loads may lead to the generation and expansion of surface cracks, such as the cracks observed in the Zhuanglanghe aqueduct^3^ and the Huojia arch dam in China^4^, which threatens the safety of these structures. Proper evaluation of the temperature load in design could improve the safety of concrete structures. At present, the temperature load design codes are gradually evolving in various countries. For example, the determination method of the temperature load is optimized in the AASHTO LRFD bridge design specifications^5^ based on the studies conducted by Potgieter and Gamble^6^, Imbsen and Vandershaf^7^, and Roeder^8^,^9^, and the optimization makes the designed temperature load more adaptable for the local climate.

Field measurements^10–14^ and numerical simulations^15–17^ are two effective ways to investigate the thermal effect. Experiments are necessary for obtaining the real thermal responses of concrete structures. For example, Lu et al.^18^ studied the impact of solar radiation and extreme thermal effects on concrete box girder bridges via field observations, and Chang^19^ investigated the thermal behavior of composite box-girder bridges subjected to environmental factors based on a 20-month statistical analysis of measured data. Moreover, numerical simulation is a powerful tool for predicting the thermal effect of concrete structures. A finite element model considering solar radiation, air temperature, and longwave radiation was established to predict concrete temperature changes by Larsson et al.^17^. An efficient vertical discrete model was established for the efficient numerical calculation of concrete temperatures by Fan et al.^20^, and the predicted temperatures matched well with the measured values. Shi et al.^21^ and Lin et al.^22^,^23^ used both methods to reveal the influence of temperature distribution on various concrete structures. Usually, it is more convenient to evaluate the temperature distribution by numerical simulation than by long-term field measurement since numerical simulation is repeatable and applicable in the design phase. Among all the numerical methods, the finite element method is the most popular and suitable method for predicting the temperature field of complex concrete structures. Some simple and specific problems^20^,^24^ could also be solved by an analytical method.

The temperature field of concrete structures can be affected by various environmental factors, and some necessary environmental factors, such as solar radiation, air convection, and heat conduction, have been widely considered in the prediction of concrete temperature^25–27^. Zhang et al.^28^ conducted wind tunnel tests on the convective heat transfer coefficient of concrete surfaces and proposed a relationship between the convective heat transfer coefficient and the wind speed. Sheng et al.^29^ investigated the influence of solar radiation absorption on the temperature field of concrete box girders. Various solar radiation models have also been developed in the field of meteorology^30^,^31^ to consider solar radiation under different dates, times, and weather conditions. Moreover, the moisture and temperature of concrete are two mutually influential physical fields, and coupling analysis^32–34^ provides a way to accurately determine the moisture and heat field inside concrete and has been an important research topic in the field of concrete temperature.

However, longwave radiation, another important heat transfer method, has not been considered in most studies for simulating the temperature field of concrete^35–37^. Few studies consider longwave radiation as convective heat transfer with air, which improves the accuracy of simulation, but there still is room for improvement^18,38^. Currently, longwave radiation is coded in commercial finite element software, such as Ansys and Abaqus. The impact of longwave radiation has not been considered in an efficient analytical method for determining the concrete temperature due to the complex nonlinear equation of longwave radiation. Thus, it is necessary to integrate the longwave radiation effect into analytical models of concrete temperature^20^,^39^,^40^.

In this paper, a one-dimensional analytical temperature model of concrete exposed to an open-air environment was improved by introducing a linearized longwave radiation equation based on the model proposed by Fan et al.^20^. The accuracy of the improved model was verified through seven-day temperature monitoring of an open-air concrete block, which was designed as a one-dimensional temperature problem. In addition, the variation characteristics of the heat energy of the concrete were extracted, decomposed and analyzed.

## Theoretical model

### Basic hypothesis

**Figure 1:**
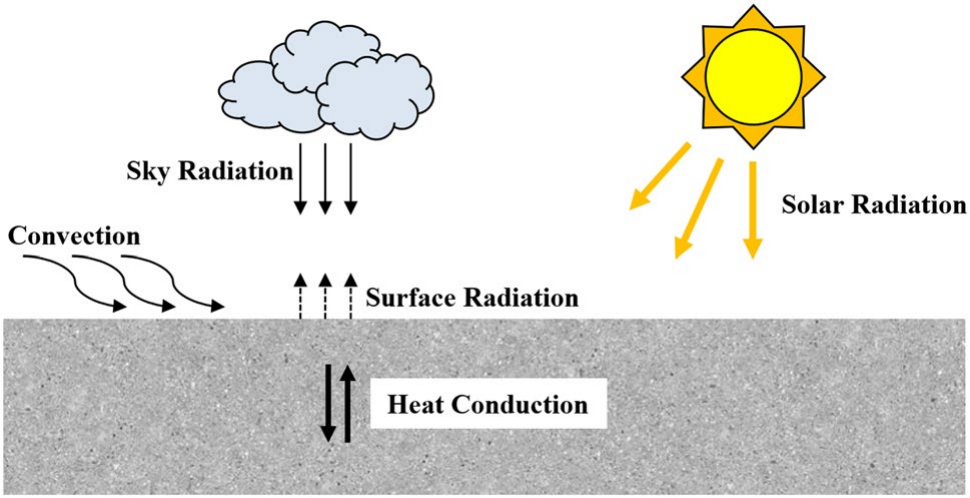
Illustration of heat exchange in open-air concrete.

The model for calculating the one-dimensional transient temperature of concrete structures is improved by considering several main environmental factors, including solar radiation, convection and longwave radiation between the sky and concrete surfaces (Fig. 1). These factors affect the temperature of the concrete surface and subsequently affect the heat conduction process and temperature distribution in the concrete. By introducing each single equation between every environmental factor and the concrete temperature behavior into the joint equation, the whole process of the concrete temperature field under various environmental factors can be solved.

### Model of heat exchange

The heat exchange between concrete and the environment only occurs through the surface. For a micro surface on an open-air concrete surface of area *S*, the total heat exchange *q*_e_ with the environment consists of three components: *q*_s_ caused by solar radiation, *q*_r_ caused by longwave radiation, and *q*_c_ caused by convection:

1 $$ q_{{\text{e}}} = q_{{\text{s}}} + q_{{\text{r}}} + q_{{\text{c}}} $$

The heat from solar radiation is not completely absorbed by the concrete due to reflection on its surface. Thus, the heat absorbed by concrete from solar radiation can be expressed as^41^:

2 $$ q_{{\text{s}}} = \alpha \cdot I \cdot S $$

where *α* is the absorptivity and *I* is the total solar radiation on the concrete surface (W/m^2^).

Convection is the heat exchange between air and the concrete surface and can be described by Newton’s cooling law as^42^:

3 $$ q_{{\text{c}}} = h_{{\text{c}}} \cdot (T_{{{\text{air}}}} - T_{{\text{s}}} ) \cdot S $$

where *h*_c_ is the convection heat transfer coefficient between the concrete and air (W/m^2^/℃), and *T*_air_ is the air temperature.

Longwave radiation is a media-free way of exchanging heat between two objects. The heat exchange caused by longwave radiation between the open-air concrete surface and the sky is expressed as^43^,^44^:

4 $$ q_{{\text{r}}} = q_{sky} - q_{s} = \varepsilon \sigma [(T_{{{\text{sky}}}} + 273.15)^{4} - (T_{{\text{s}}} + 273.15)^{4} ] \cdot S $$

where *q*_sky_ is the longwave radiation from the concrete, *q*_s_ is the longwave radiation from the sky, $\varepsilon$ is the emissivity, $\sigma$ is the Stefan-Boltzmann constant, $\sigma = 5.670367 \times 10^{ - 8} ({\text{W}} \times {\text{m}}^{{ - 2}} \times K^{{ - 4}} )$, *T*_s_ is the temperature of the concrete surface, and *T*_sky_ is the equivalent temperature of the sky.

According to the intermediate value theorem, Eq. (4) can be linearized as:

5 $$ \begin{aligned} q_{{\text{r}}} & = 4\varepsilon \sigma (T_{\eta } + 273.15)^{3} \cdot (T_{{{\text{sky}}}} - T_{{\text{s}}} ) \cdot S \\ & \approx 4\varepsilon \sigma (T_{{{\text{sky}}}} + 273.15)^{3} \cdot (T_{{{\text{sky}}}} - T_{{\text{s}}} ) \cdot S \\ & = h_{{\text{ r}}} \cdot (T_{{{\text{sky}}}} - T_{{\text{s}}} ) \cdot S \\ \end{aligned} $$

where $T_{\eta }$ is a variable between $T_{{{\text{sky}}}}$ and $T_{{\text{s}}}$, and $h_{{\text{ r}}}$ is the equivalent heat transfer coefficient (W/m^2^/℃) of longwave radiation.

### Model of heat conduction

The heat conduction in concrete follows Fourier's law. If *k* indicates the thermal conductivity (W/m/℃), the conducted heat *q*_k_ per unit area is proportional to the temperature gradient $\Delta T$^44^:

6 $$ q_{{\text{k}}} = k\Delta T = k\left( {\frac{\partial T}{{\partial x}}\overrightarrow {{i_{x} }} + \frac{\partial T}{{\partial y}}\overrightarrow {{i_{y} }} + \frac{\partial T}{{\partial z}}\overrightarrow {{i_{z} }} } \right) $$

For a one-dimensional concrete model with a height of *H*, the temperature is one-dimensionally distributed along the height, and Eq. (6) can be simplified as:

7 $$ q_{{\text{k}}} = k\frac{dT}{{dH}} $$

### Model solution

**Figure 2:**
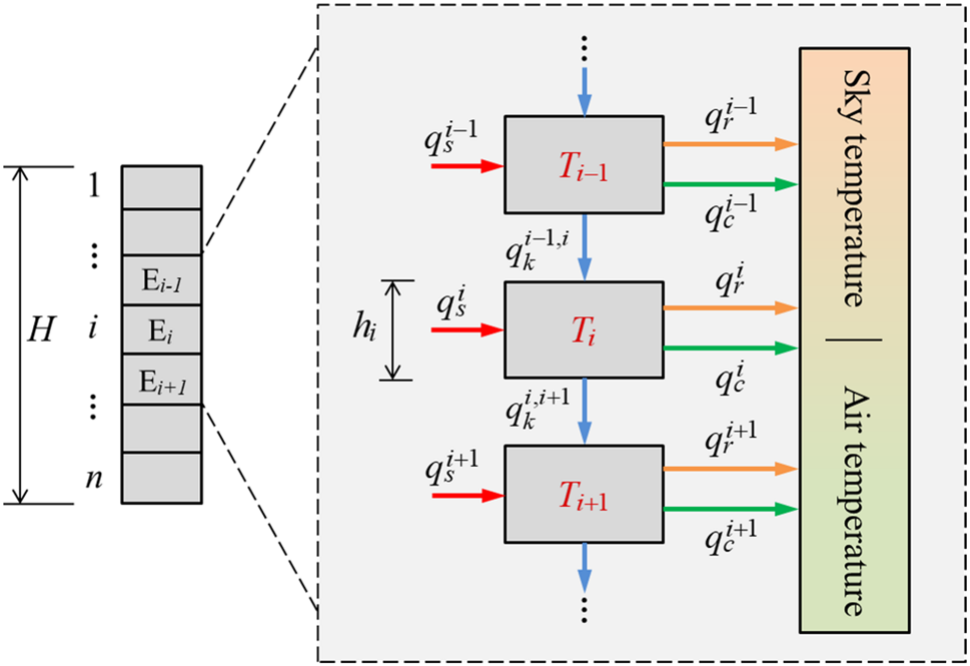
Division of concrete elements and illustration of heat exchanges.

A concrete column is divided into *n* one-dimensional elements, and the material is homogenous (Fig. 2). $q_{{\text{s}}}^{i}$, $q_{{\text{r}}}^{i}$, and $q_{{\text{c}}}^{i}$ indicate the heat changes in the *i*_th_ (*i* = 1, 2, …, n) element caused by solar radiation, longwave radiation, and convection, respectively. $q_{{\text{k}}}^{i,i + 1}$ is the heat conduction from the *i*_th_ element to the (*i* + 1)_th_ element. The inflow heat is defined as the positive heat for all the components.

According to Eqs. (2–5) and (7), these heat components of the *i*_th_ element are expressed as:

8 $$ \left\{ \begin{gathered} q_{{\text{s}}}^{i} = \alpha_{i} S_{{\text{s}}}^{i} I_{i} \hfill \\ q_{{\text{r}}}^{i} \approx h_{{\text{r}}}^{i} S_{{\upvarepsilon }}^{i} (T_{{{\text{sky}}}} - T_{i} ) \hfill \\ q_{{\text{c}}}^{i} = h_{{\text{c}}}^{i} S_{{\text{c}}}^{i} (T_{{{\text{air}}}} - T_{i} ) \hfill \\ q_{{\text{k}}}^{i,i + 1} \approx k_{i + 1} \frac{{T_{i} - T_{i + 1} }}{{h_{i + 1} }} = \frac{{k_{i + 1} }}{{h_{i + 1} }}(T_{i} - T_{i + 1} ) \hfill \\ \end{gathered} \right. $$

According to the law of energy conservation, the internal energy change of the *i*_th_ (*i* = 2, 3, …, n−1) element per unit time is equal to the sum of all types of heat changes per unit time:

9 $$ \begin{aligned} c_{i} m_{i} \frac{{dT_{i} }}{dt} & = q_{{\text{k}}}^{i - 1,i} - q_{{\text{k}}}^{i,i + 1} + q_{{\text{s}}}^{i} + q_{{\text{r}}}^{i} + q_{{\text{c}}}^{i} \\ & = \frac{{k_{i} }}{{h_{i} }}(T_{i - 1} - T_{i} ) - \frac{{k_{i + 1} }}{{h_{i + 1} }}(T_{i} - T_{i + 1} ) + \alpha_{i} S_{{\text{s}}}^{i} I_{i} + h_{{\text{ r}}} S_{{\text{r}}}^{i} (T_{{{\text{sky}}}} - T_{i} ) + h_{{\text{c}}} S_{{\text{c}}}^{i} (T_{{{\text{air}}}} - T_{i} ) \\ & = \frac{{k_{i} }}{{h_{i} }}T_{i - 1} + \left( { - \frac{{k_{i + 1} }}{{h_{i + 1} }} - \frac{{k_{i} }}{{h_{i} }} - h_{{\text{ r}}} S_{{\text{r}}}^{i} - h_{{\text{c}}} S_{{\text{c}}}^{i} } \right)T_{i} + \frac{{k_{i + 1} }}{{h_{i + 1} }}T_{i + 1} + \alpha_{i} S_{{\text{s}}}^{i} I_{i} + h_{{\text{r}}} S_{{\text{r}}}^{i} T_{{{\text{sky}}}} + h_{{\text{c}}} S_{{\text{c}}}^{i} T_{{{\text{air}}}} \\ & = U_{{\text{k}}}^{i} T_{i - 1} - (U_{{\text{k}}}^{i + 1} + U_{{\text{k}}}^{i} + U_{{\text{r}}}^{i} + U_{{\text{c}}}^{i} )T_{i} + U_{{\text{k}}}^{i + 1} T_{i + 1} + \alpha_{i} S_{{\text{s}}}^{i} I_{i} + h_{{\text{r}}} S_{{\text{r}}}^{i} T_{{{\text{sky}}}} + h_{{\text{c}}} S_{{\text{c}}}^{i} T_{{{\text{air}}}} \\ \end{aligned} $$

where *c*_*i*_ is the specific heat capacity of the *i*_th_ element and *m*_*i*_ is the mass of the *i*_th_ element.

Specifically, the internal energy changes of the top and bottom elements are:

10 $$ c_{1} m_{1} \frac{{dT_{1} }}{dt} = - (U_{k}^{2} + U_{r}^{1} + U_{c}^{1} )T_{1} + U_{{\text{k}}}^{2} T_{2} + \alpha_{1} S_{{\text{s}}}^{1} I_{1} + h_{{\text{r}}} S_{{\text{r}}}^{1} T_{{{\text{sky}}}} + h_{{\text{c}}} S_{{\text{c}}}^{1} T_{{{\text{air}}}} $$

11 $$ c_{n} m_{n} \frac{{dT_{n} }}{dt} = U_{{\text{k}}}^{n} T_{n - 1} - (U_{{\text{k}}}^{n} + U_{{\text{r}}}^{n} + U_{{\text{c}}}^{n} )T_{{\text{n}}} + \alpha_{n} S_{{\text{s}}}^{n} I_{n} + h_{{\text{r}}} S_{{\text{r}}}^{n} T_{{{\text{sky}}}} + h_{{\text{c}}} S_{{\text{c}}}^{n} T_{{{\text{air}}}} $$

Equations (9–11) can be expressed by the differential equation $\frac{dx}{{dt}} = Ax + Bu$, where:

12 $$ A = \left[ {\begin{array}{*{20}l} { - \frac{{U_{{\text{k}}}^{2} + U_{{\text{r}}}^{{1}} + U_{{\text{c}}}^{{1}} }}{{c_{1} m_{1} }}} & {\frac{{U_{{\text{k}}}^{{2}} }}{{c_{1} m_{1} }}} & {} & {} & {} \\ {\frac{{U_{{\text{k}}}^{{2}} }}{{c_{2} m_{2} }}} & { - \frac{{U_{{\text{k}}}^{3} + U_{{\text{k}}}^{{2}} + U_{{\text{r}}}^{{2}} + U_{{\text{c}}}^{{2}} }}{{c_{2} m_{2} }}} & {\frac{{U_{{\text{k}}}^{3} }}{{c_{2} m_{2} }}} & {} & {} \\ {} & {} & \ddots & {} & {} \\ {} & {} & {\frac{{U_{{\text{k}}}^{n - 1} }}{{c_{n - 1} m_{n - 1} }} \, } & { - \frac{{U_{{\text{k}}}^{n} + U_{{\text{k}}}^{n - 1} + U_{{\text{r}}}^{n - 1} + U_{{\text{c}}}^{n - 1} }}{{c_{n - 1} m_{n - 1} }}} & {\frac{{U_{{\text{k}}}^{n} }}{{c_{n} m_{n} }}} \\ {} & {} & {} & {\frac{{U_{{\text{k}}}^{n} }}{{c_{n} m_{n} }}} & { - \frac{{U_{{\text{k}}}^{n} + U_{{\text{r}}}^{n} + U_{{\text{c}}}^{n} }}{{c_{n} m_{n} }}} \\ \end{array} } \right]_{n \times n} $$

13 $$ x = [T_{1} ,T_{2} ,...,T_{n} ]^{T} $$

14 $$ B = \left[ {\begin{array}{*{20}l} {\frac{{\alpha_{1} S_{{\text{s}}}^{1} }}{{c_{1} m_{1} }}} & {} & {} & {} & {\frac{{U_{{\text{r}}}^{1} }}{{c_{1} m_{1} }}} & {\frac{{U_{{\text{c}}}^{1} }}{{c_{1} m_{1} }}} \\ {} & {\frac{{\alpha_{2} S_{{\text{s}}}^{2} }}{{c_{2} m_{2} }}} & {} & {} & {\frac{{U_{{\text{r}}}^{{2}} }}{{c_{2} m_{2} }}} & {\frac{{U_{{\text{c}}}^{{2}} }}{{c_{2} m_{2} }}} \\ {} & {} & \ddots & {} & \vdots & \vdots \\ {} & {} & {} & {\frac{{\alpha nS_{{\text{s}}}^{n} }}{{c_{n} m_{n} }}} & {\frac{{U_{{\text{r}}}^{n} }}{{c_{n} m_{n} }}} & {\frac{{U_{{\text{c}}}^{n} }}{{c_{n} m_{n} }}} \\ \end{array} } \right]_{n \times (n + 2)} $$

15 $$ u = [I_{1} ,I_{2} ,...,I_{n} ,T_{{{\text{sky}}}} ,T_{{{\text{air}}}} ]^{T} $$

After the initial temperature field of concrete is given, the variation in the concrete’s temperature field with time under the influences of various environmental factors can be solved by $\frac{dx}{{dt}} = Ax + Bu$. It should be noted that the method proposed by Fan et al.^20^ is improved by introducing longwave radiation to matrix *B* and a variable sky temperature to vector *u* in this article.

## Experiment on an open-air concrete block

To verify the accuracy of the improved analytical model, a concrete block was placed in an open-air environment, and its temperature distribution and several environmental factors were measured.

### Test design and process

**Figure 3:**
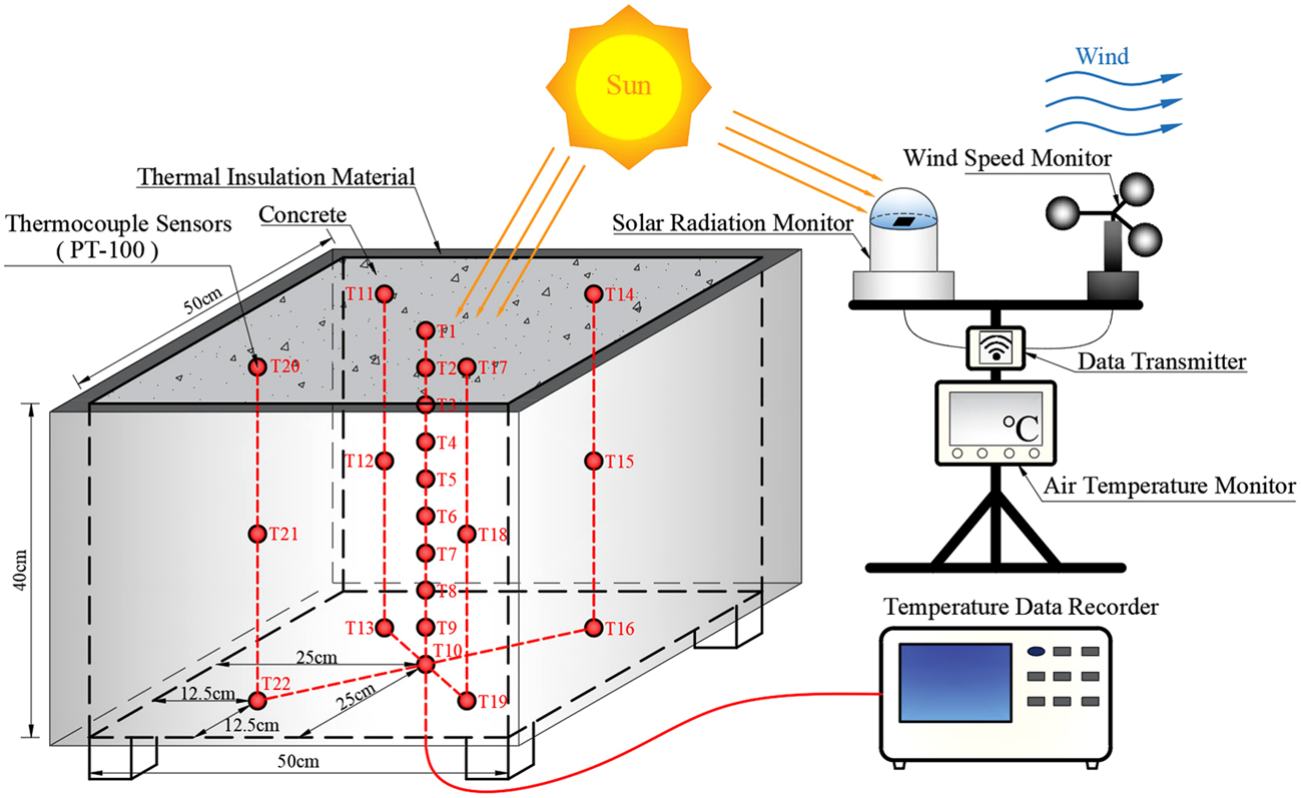
Sketch of the experimental system including the concrete block and several sensors.

The concrete specimen is a cuboid block with a size of 50 cm × 50 cm × 40 cm (Fig. 3). The material components and their proportions in the concrete are listed in Table 1. To maintain the same convection conditions on the top and bottom surfaces of the concrete block, the block was lifted by placing four small wood blocks below its four bottom corners. Its lateral surfaces were covered by a layer of thermal insulation material with a thickness of 3 cm and a thermal conductivity of 0.03 W/℃/m^45^. Because the thermal conductivity of the studied concrete is approximately 3 W/℃/m, the layer of thermal insulation material can be thermally equivalent to a layer of concrete that has a thickness of 3 m. Therefore, the temperature field of the concrete block was designed as a one-dimensional problem in the vertical direction and is suitable for verifying the improved analytical model.

**Table 1:** Materials components and their proportions in the studied concrete.

Water-cement ratio	Aggregate-cement ratio	Sand ratio	Material content/(kg m^−3^)				
			Water	Cement	Fine aggregate	Coarse aggregate	Fly ash
0.31	3.78	39%	166	477	703.25	1101.75	52

Before concreting, ten thermocouple sensors (T1–T10) were installed from top to bottom in the center of the concrete to measure the vertical distribution of its temperature field. In addition, twelve other thermocouple sensors (T11–T22) were placed in the top, middle, and bottom horizontal layers, respectively, to examine the temperature differences in the horizontal section. The PT100 thermocouple sensor has a measurement accuracy of 0.1 °C. The data of all thermocouple sensors were automatically recorded and stored by a data recorder.

Moreover, solar radiation, wind speed and air temperature were also measured and recorded by the corresponding monitors. The measurement accuracies of the solar radiation monitor, the wind speed monitor, and the air temperature monitor are 0.1 W/m, 0.1 m/s, and 0.1 ℃, respectively. The distances between all the monitors and the concrete specimen were less than 5 m to ensure the reliable acquisition of environmental factors. All the data were automatically collected at intervals of 300 s.

The experiment was conducted at an open-air site on the Jiang'an Campus of Sichuan University (30.55° N, 104.00° E), and there is no high building around the site. The concreting date was March 13, 2022, and curing methods, including cover and water sprinkling, were applied until the age of 28 days. The concrete temperature and environmental factors were observed for 7 days from 00:00 on May 1, 2022, to 00:00 on May 8, 2022, and the weather during the period was sunny.

### Measured concrete temperature and environmental factors

**Figure 4:**
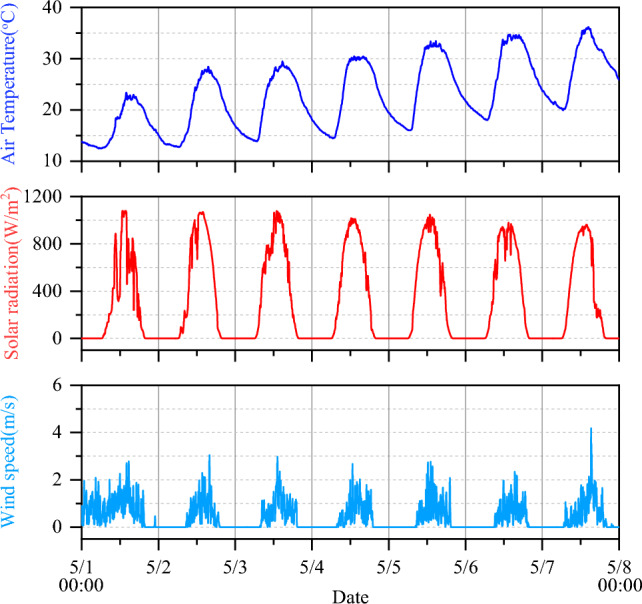
The data of environmental factors during the test.

Figure 4 shows the air temperature, solar radiation, and wind speed over time during the test period. The air temperature, solar radiation, and wind speed fluctuate in a daily cycle. On continuous sunny days, the air temperature generally increased during the test period. The instantaneous fluctuation in the wind speed is significant, and its average value is 0.5 m/s. Generally, the environmental data measured in the test are normal and applicable.

**Figure 5:**
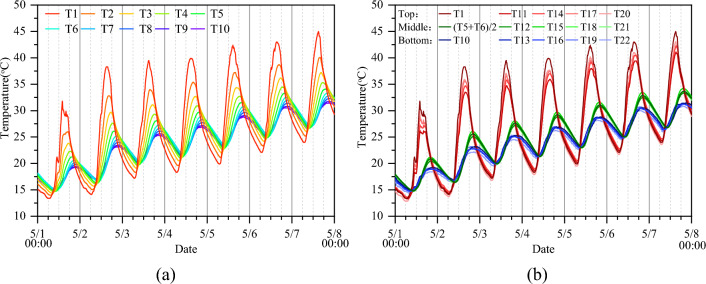
Temperature variations measured by various sensors in the concrete during the test. (**a**) Temperature variations measured in the centers of various horizontal sections and (**b**) comparison of temperatures measured at the central and peripheral points in the top, middle, and bottom sections.

Figure 5 shows the temperature variations measured by various sensors in the concrete specimen during the test. The temperature variations measured in the centers of various horizontal sections are shown in Fig. 5a. The figure shows that the measured temperature varies significantly in the vertical direction. The temperatures at the top and bottom positions are the highest and lowest, respectively, during the day, and their difference can reach over 18.0 °C. However, the temperatures measured in the same horizontal section are almost the same (Fig. 5b). For example, the temperature differences in the top, middle, and bottom sections are less than 1.0 ℃, 1.0 ℃, and 4.0 ℃, respectively, which are negligible compared to the measured results in the vertical direction. In general, it is appropriate to assume that the temperature distribution of the concrete specimen is one-dimensional in the vertical direction. Therefore, only the data measured by the T1–T10 sensors were used in the following analyses.

### Hysteresis effect of temperature change

**Figure 6:**
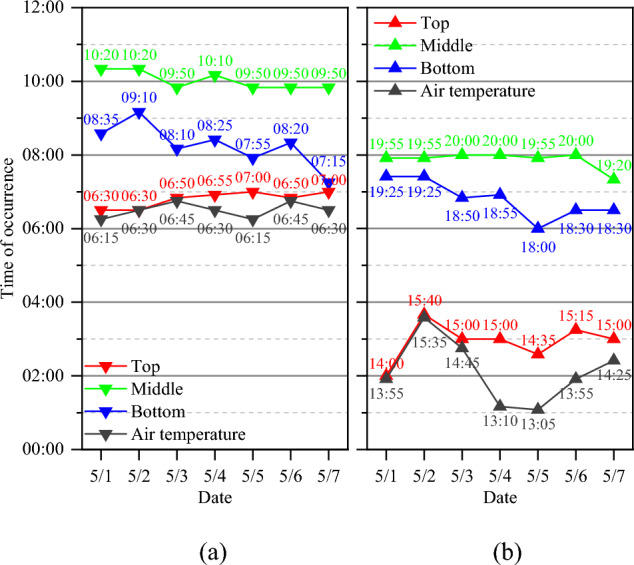
Occurrence times of (**a**) the minimum and (**b**) the maximum temperatures in a day at different heights.

The occurrence times of the minimum and maximum temperatures in a day at different heights were analyzed (Fig. 6). These extreme temperatures in the concrete block occurred after the occurrence of the extreme air temperature. The minimum temperatures in a day always occurred in the morning, and the average hysteretic times of the minimum temperature at the top, bottom and middle of the concrete block were 0.28, 1.75, and 3.52 h, respectively. The maximum temperatures in a day always occurred in the afternoon, and the average hysteretic times of the maximum temperature at the top, bottom and middle of the concrete block were 0.80, 4.67, and 5.75 h, respectively. The concrete temperature indeed changes after the air temperature does, and the concrete top surface is most sensitive to changes in the air temperature. Since the top surface experiences both the highest and the lowest temperatures in the concrete block (Fig. 5), it must be subjected to additional heat absorption and dissipation compared with the other surfaces.

### Distribution pattern of the concrete temperature

**Figure 7:**
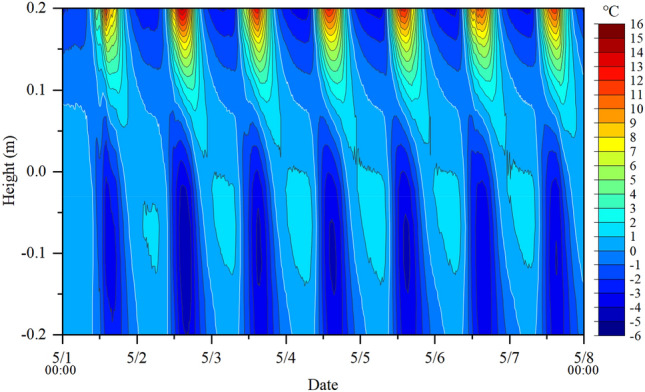
Variation in the relative temperature with time on the vertical line in the center of the concrete block.

A nonlinear temperature gradient is a significant thermal load recommended by AASHTO^5^, so it is important to investigate the temperature distribution in the vertical direction. To facilitate comparisons among the temperature distributions obtained at various times, the relative temperature instead of the monitored temperature is plotted in Fig. 7, in which the relative temperature is defined as the temperature difference between the monitored and the average temperatures of all monitoring points on the vertical line in the center of the concrete block. The figure shows that, under the influence of various environmental factors, the relative temperature at an arbitrary location could be positive for a period of time and become negative at other times. In general, the maximum positive relative temperature can reach 11–15 ℃ and occurs at the top of the concrete, and the maximum negative one can reach − 3 ~ − 5 ℃ and occurs at a height of 0.1 m instead of at the bottom.

**Figure 8:**
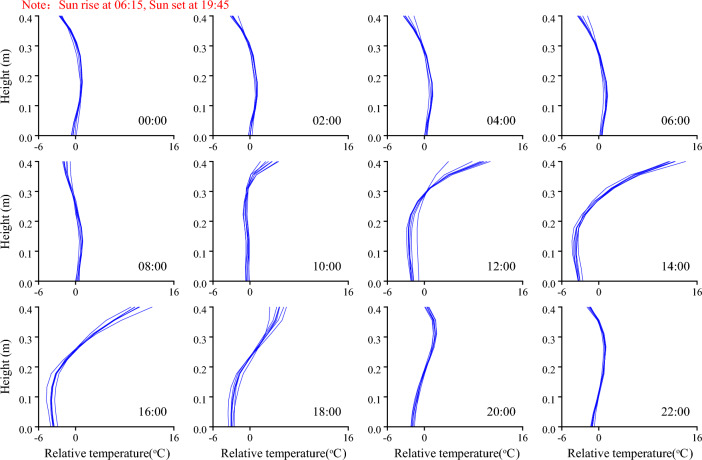
Distribution patterns of the relative temperature at various times.

The distributions of the relative temperature at various times of the day are shown in Fig. 8 to illustrate the distribution pattern of the concrete temperature more clearly. The distribution pattern of the concrete temperature varies with time and can generally be classified into two categories. The first pattern of temperature distribution is similar to a left parenthesis, and it can be named a positive pattern and often occurs from 12:00 to 16:00. The second pattern is similar to a right parenthesis, and it can be named a negative pattern and often occurs from 22:00 to 06:00 + 1. The first pattern has a higher relative temperature on the top of the concrete block under the influence of strong solar radiation in the afternoon, and the second pattern has a lower relative temperature on the top of the concrete block under the influence of extra heat dissipation from longwave radiation. In addition, although the distribution pattern of temperature changes significantly throughout the day, the distribution patterns measured at the same time on different dates are almost the same during the observation period. The air temperature measured at the same time increases daily (Fig. 4), but it does not affect the distribution pattern of the relative temperature. However, the solar radiation measured at the same time does not change much and results in the same distribution pattern of the relative temperature. Thus, the air temperature is highly related to the average temperature of concrete, and solar radiation is the main factor that determines the distribution pattern and gradient of the temperature.

**Figure 9:**
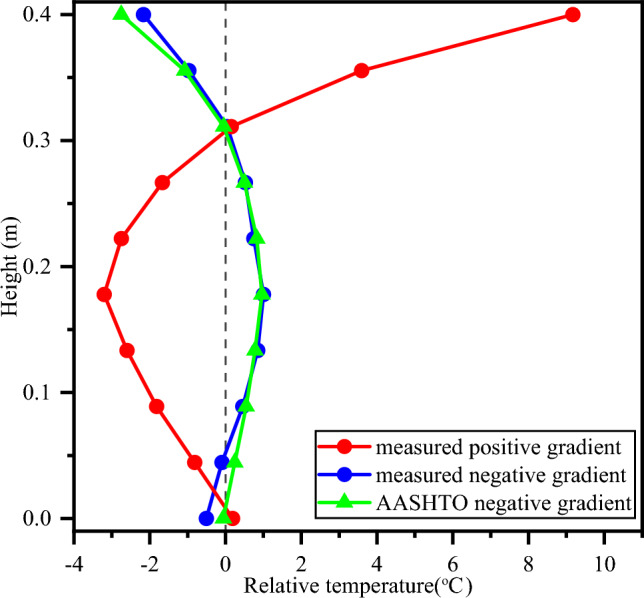
Comparison of temperature distributions measured in the experiment and suggested by the AASHTO.

Compared with the relative temperature at the bottom of the concrete block, the first and second patterns of temperature distribution could also be characterized as positive and negative relative temperatures at the top of the concrete block, respectively, so they have positive and negative gradients from the bottom to the top of the concrete block, respectively. The maximum positive gradient is observed at 14:00, and the maximum negative gradient is observed at 2:00 (Fig. 9). The absolute value of the minimum relative temperature is approximately 0.3 times that at the top. Since the relative temperature is approximately 0 ℃ at the bottom, the negative temperature gradient is approximately 0.3 times the positive one between the top and the bottom. This finding verifies a specification in AASHTO^5^ that a negative temperature gradient can be calculated as 0.3 times the positive one. In addition, the calculated relative temperature distribution with the suggested negative gradients in the specification is nearly identical to the measured relative temperature distribution (Fig. 9).

## Model verification and energy analysis

### Material parameters and numerical model

The temperature distribution along the vertical direction of the concrete specimen during the test is calculated by the differential form of the improved concrete temperature model. Based on the research of many scholars on concrete thermal parameters^28^,^45^,^46^, appropriate homogeneous isotropic thermal parameters were adopted for the following analysis, as listed in Table 2.

**Table 2:** Thermal parameters of the concrete.

Specific heat (kJ/kg)	Density (kg/m^3^)	Thermal conductivity (kJ/h/m/℃)	Convective coefficient (kJ/h/m^2^/℃)	Absorptivity	Emissivity
1.03	2500	10.8	30	0.5	0.9

The boundary conditions, including solar radiation, air convection, and longwave radiation, were set on the top surface, air convection was set on the bottom surface, and adiabatic boundaries were set on the lateral surfaces.

The concrete specimen is evenly divided into 20 elements from the top to the bottom, and these elements have a height of *h* = 0.02 m and a width of *b* = 0.5 m. Therefore, $U_{k}^{i} = k_{i} /h_{i} = U_{k} = k/h$, and matrices ***A*** and ***B*** of the improved model can be expressed as follows:

16 $$ A = \left[ {\begin{array}{*{20}l} { - \frac{{U_{k} + U_{r}^{1} + U_{c}^{1} }}{{c_{1} m_{1} }}} & {\frac{{U_{k} }}{cm}} & {} & {} & {} \\ {\frac{{U_{k} }}{cm}} & 0 & {\frac{{U_{k} }}{cm}} & {} & {} \\ {} & {} & \ddots & {} & {} \\ {} & {} & {\frac{{U_{k} }}{cm}} & 0 & {\frac{{U_{k} }}{cm}} \\ {} & {} & {} & {\frac{{U_{k} }}{cm}} & { - \frac{{U_{k} + U_{c}^{20} }}{cm}} \\ \end{array} } \right]_{20 \times 20} $$

17 $$ B = \left[ {\begin{array}{*{20}l} {\frac{{\alpha_{1} S_{s}^{1} }}{cm}} & {} & {} & {} & {} & {\frac{{U_{r}^{1} }}{cm}} & {\frac{{U_{c}^{1} }}{cm}} \\ {} & 0 & {} & {} & {} & 0 & 0 \\ {} & {} & \ddots & {} & {} & \vdots & \vdots \\ {} & {} & {} & 0 & {} & 0 & 0 \\ {} & {} & {} & {} & 0 & 0 & {\frac{{U_{c}^{20} }}{cm}} \\ \end{array} } \right]_{20 \times 22} $$

The concrete temperatures measured at 00:00 on May 1st were taken as the initial temperature of these elements, and the monitored solar radiation and air temperature were input as transient boundary conditions. The *T*_sky_ of longwave radiation was set to *T*_air_—11 °C, as suggested by ISO 13,790 RMAT^47^. The time step of the transient analysis was set as 300 s, and the LSIM function in MATLAB was used to solve the equation $\frac{dx}{{dt}} = Ax + Bu$.

### Calculation results of the concrete temperature

**Figure 10:**
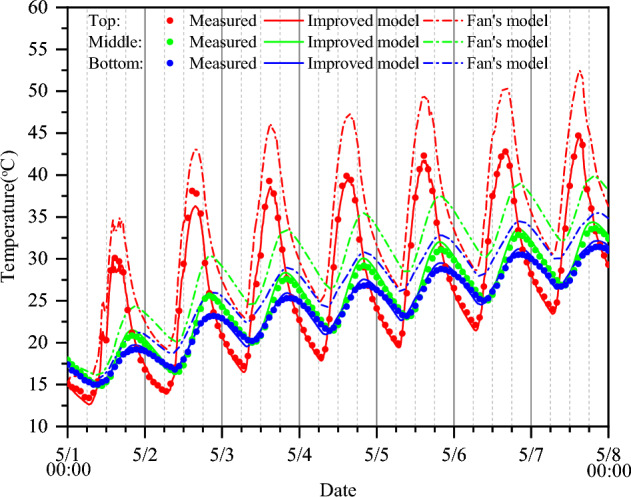
Comparisons of the measured temperatures with the calculated temperatures of the improved model and Fan’s model.

Figure 10 shows the calculated temperatures of the improved model and Fan’s model. The temperatures calculated by the improved model that integrates longwave radiation match the measured temperature better than those calculated by Fan’s model. By neglecting the long-wave radiation, Fan’s model overestimates the concrete temperature, and the calculated temperature at the top is always higher than that at the bottom. However, as demonstrated by both the experimental observations and the improved model, under the influence of longwave radiation, the temperature at the top could decrease rapidly at night and even become lower than the minimum temperature at the bottom.

### Decomposition and analysis of concrete heat energy

**Figure 11:**
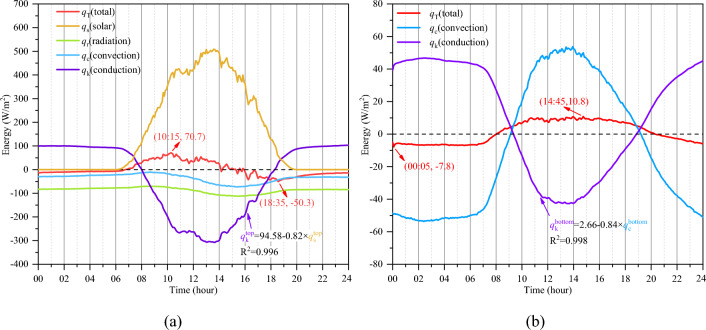
Heat energy variations of (**a**) the top element and (**b**) the bottom element of the concrete model.

An additional advantage of temperature numerical calculations is that each heat component can be decomposed from the concrete temperature. In the improved analytical model, the total heat flowing in or out of a concrete element $q_{{\text{T}}}$ consists of four components: the heat energy caused by solar radiation, longwave radiation, convection, and conduction, expressed as $q_{{\text{s}}} ,q_{{\text{r }}} ,q_{{\text{c}}} ,q_{{\text{k}}}$, respectively. The heat energy variations of the top and bottom elements obtained at the same time but from different dates are averaged, and their variations in a daily cycle are shown in Fig. 11a, b, respectively. Positive and negative values represent heat inflow and outflow, respectively, and the superscript represents the element position in the following analysis.

As shown in Fig. 11a, each energy component of the top element fluctuates in a daily cycle. The solar radiation energy $q_{{\text{s}}}^{{{\text{top}}}}$ is the strongest energy component among all the components of the top element, and $q_{{\text{s}}}^{{{\text{top}}}}$ always increases to its peak value (approximately 509.0 W/m^2^) at 13:00–14:00. Other energy components of the top element, especially the conduction energy $q_{{\text{k}}}^{{{\text{top}}}}$, vary with changes in solar radiation. There is a significant negative correlation between $q_{{\text{k}}}^{{{\text{top}}}}$ and $q_{{\text{s}}}^{{{\text{top}}}}$, which is fitted as $q_{{\text{k}}}^{{{\text{top}}}} = 94.58 - 0.82q_{{\text{s}}}^{{{\text{top}}}}$. Thus, solar radiation controls the temperature of a surface exposed to the sun.

Since the concrete bottom surface is not exposed to sunlight, the total energy of the bottom element consists of only the convection energy $q_{{\text{c}}}^{{{\text{bottom}}}}$ and the conduction energy $q_{{\text{k}}}^{{{\text{bottom}}}}$ (Fig. 11b). There is also a significant negative correlation between $q_{{\text{k}}}^{{{\text{bottom}}}}$ and $q_{{\text{c}}}^{{{\text{bottom}}}}$, which is fitted as $q_{{\text{k}}}^{{{\text{bottom}}}} = 2.66 - 0.84q_{{\text{c}}}^{{{\text{bottom}}}}$. Convection is the main reason for the temperature variation in a shaded surface.

**Figure 12:**
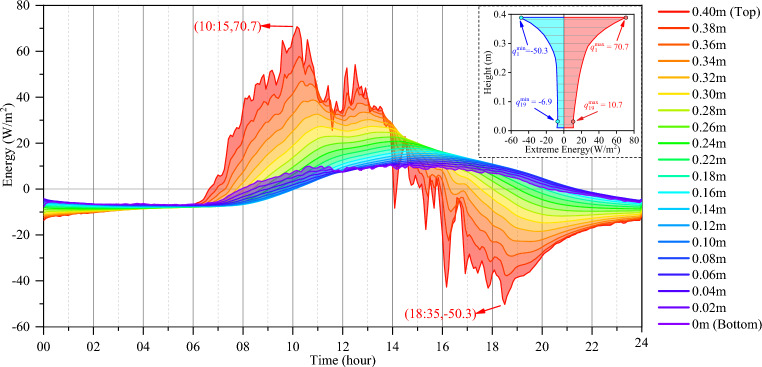
Variations in the total heat energy at different heights.

The total heat energies at different heights of the concrete block obtained at the same time but from different dates are also averaged, and their variations in a daily cycle are shown in Fig. 12. The variations in the total heat energy at various heights fluctuate throughout the day with different amplitudes. The top surface has the largest peak and trough amplitudes of the total heat energy. The maximum inflow energy of the top surface is 70.7 W/m^2^, and the maximum outflow energy of the top surface is − 50.3 W/m^2^. The variation in the total energy at a height of 0.02 m is the smallest, which is approximately 1/7 of that at the top surface. Except for the bottom surface, the location closer to the top surface is more susceptible to the external environment, and its total heat energy fluctuates more obviously during the daily cycle. In addition, the change in total heat energy at different elevation positions has a hysteresis effect, as analyzed in Section “Hysteresis effect of temperature change”. Thus, except for the bottom surface, the location further away from the top surface has a more significant hysteresis effect on the change in total heat energy.

## Conclusion

By considering solar radiation, thermal convection, thermal conduction and especially longwave radiation, an improved analytical model of concrete temperature is proposed and verified by monitoring the temperature of an open-air concrete block. In addition, the energy characteristics of the open-air concrete were analyzed by an improved analytical model. The following conclusions can be drawn.

1. Under the influence of an open-air environment, the temperature change in concrete exhibits a hysteresis effect in which extreme temperatures in concrete occur after the extreme air temperature occurs. In addition, the transient distribution of the concrete temperature on the vertical line in the center of the concrete block varies with time and can be classified into two categories, which have overall positive and negative gradients from the bottom to the top, respectively. The monitored temperatures show that the maximum overall negative gradient is approximately 0.3 times the maximum overall positive gradient, and this observation provides further evidence for a specification of the temperature gradient suggested by the AASHTO.
2. Under the influence of longwave radiation, the temperature at the top of the concrete block decreases rapidly at night and even becomes lower than the minimum temperature at the bottom. After considering the effect of longwave radiation, the improved analytical model could result in a transient variation in temperature that matches the monitoring temperature of the open-air concrete block very well. However, an analytical model that does not include the effect of longwave radiation could overestimate the temperature of open-air concrete.
3. The heat energy analysis results indicate that solar radiation and thermal convection control the transient variation in heat energy on the sunlit surface and the backlit surface of open-air concrete, respectively. In addition, the hysteresis effect also exists in the transient change in the total heat energy at different heights of the concrete block, and the location further away from the top surface has a more significant hysteresis effect on the change in total heat energy.

## Data Availability

The datasets used in the current study are available from the first author upon reasonable request.
